# Curriculum Gaps in Periodontal Knowledge Among Medical Students: A Cross‐Sectional Study

**DOI:** 10.1111/eje.70132

**Published:** 2026-03-10

**Authors:** Atanur Sarioglu, Tugrul Kirtiloglu

**Affiliations:** ^1^ Department of Periodontology, Faculty of Dentistry Ondokuz Mayis University Samsun Turkey

**Keywords:** medical curriculum, medical students, oral health behaviour, periodontal disease

## Abstract

**Introduction:**

Periodontal disease is a major public health concern with well‐established systemic implications. Despite the increasing emphasis on the periodontal‐systemic health connection, the extent to which periodontal disease is integrated into medical education is still unclear. This study aims to assess the knowledge, oral hygiene practices and attitudes of first‐year and final‐year medical students regarding periodontal disease, while also evaluating its inclusion in the medical curriculum.

**Materials and Methods:**

A cross‐sectional study was conducted among first‐ and final‐year medical students at Ondokuz Mayis University Faculty of Medicine, Samsun, Turkey. Data were collected using a self‐administered questionnaire comprising 32 items across four domains: oral hygiene behaviours, periodontal education, knowledge of periodontal disease and awareness and attitudes toward periodontal health. Data were analysed using IBM SPSS v.21.

**Results:**

A total of 596 medical students participated in the study, consisting of 281 first‐year and 315 final‐year students. Final‐year students reported significantly higher rates of receiving periodontal education during medical school (41.3%) compared to first‐year students (22.4%). In four out of five knowledge‐based questions, final‐year students demonstrated significantly higher correct response rates than first‐year students (*p* < 0.001). Additionally, in the attitude and awareness section, final‐year students showed significantly greater agreement with statements linking periodontal disease to diabetes (*p* < 0.001).

**Conclusions:**

Although final‐year students performed significantly better than first‐year students in both knowledge and awareness regarding periodontal health, the overall level of knowledge remained insufficient. These findings highlight the need for more structured and integrated periodontal education within undergraduate medical curricula to support early diagnosis, interdisciplinary collaboration and improved patient outcomes.

## 1 Introduction

Periodontal disease is a chronic, multifactorial and inflammatory condition characterised by the destruction of teeth and their supporting tissues, affecting both oral and systemic health [1]. Studies have reported that periodontal infection is not confined solely to periodontal tissues but also exerts systemic effects [2, 3]. Moreover, periodontal diseases have been shown to influence the pathogenesis and progression of various systemic conditions, including cardiovascular diseases, diabetes and pregnancy‐related complications [4, 5, 6].

These systemic effects are largely mediated through the persistent presence of pathogenic bacteria within subgingival biofilms, which trigger a sustained host inflammatory response [7]. Key bacterial components such as lipopolysaccharides (LPS) induce a pro‐inflammatory response characterised by elevated levels of cytokines such as interleukin‐1β (IL‐1β), tumour necrosis factor‐alpha (TNF‐α) and prostaglandin E2 (PGE2), which are known to drive tissue destruction and immune dysregulation [8, 9, 10]. Furthermore, activation of matrix metalloproteinases (MMPs) plays a pivotal role in the degradation of the extracellular matrix and alveolar bone loss. This pathological process directly contributes to the progression of periodontal disease [11]. Once the epithelial barrier in periodontal pockets is compromised, microbial products can enter the systemic circulation, leading to a low‐grade systemic inflammatory state [12].

Despite the well‐established links between periodontal and systemic health, the extent to which medical students are educated about periodontal disease remains unclear. Although medical professionals generally acknowledge the importance of oral health, multiple studies have shown that their understanding of periodontal disease and its systemic associations is often limited, largely due to inadequate coverage in the medical curriculum [13, 14, 15].

This study aims to evaluate the oral health behaviours, knowledge and awareness of medical students at two distinct stages of their education. By focusing on first‐year students who have just begun their training and final‐year students approaching graduation, the study examines not only expected differences in knowledge, but also whether undergraduate medical education leads to an adequate level of periodontal knowledge by the time of graduation. This approach allows for an assessment of the educational outcome of the medical curriculum with respect to periodontal and oral–systemic health, while enabling interpretation of the findings within the context of existing literature.

Unlike prior research that has centered predominantly on dental or allied health students, this study highlights an underexplored group: medical students. Assessing students at entry to medical school and those nearing graduation enables evaluation of how medical training contributes to periodontal knowledge and awareness over time, with observed knowledge levels considered in relation to previously reported findings from dental education studies. In doing so, the study seeks to identify potential gaps in curricular coverage and to provide insights that may support the integration of relevant oral health content into broader medical education frameworks.

## 2 Material and Methods

### 2.1 Study Design and Participants

First‐ and final‐year medical students from Ondokuz Mayis University Faculty of Medicine, Samsun, Turkey, were invited to participate in this study using a self‐administered structured questionnaire. In this study, ‘first‐year’ and ‘final‐year’ refer to students in the first and sixth years of a 6‐year undergraduate medical program. These two groups were selected to represent the educational extremes of the curriculum: those just entering medical school with no clinical exposure, and those nearing graduation after completing the full scope of training. This design aimed to capture differences in periodontal knowledge attributable to cumulative curricular exposure rather than transient variations in intermediate years.

The study aimed to include the entire accessible population of first‐year and final‐year medical students. All eligible students were invited to participate, and 596 of 599 students completed the questionnaire, yielding a response rate of over 99%. As a census‐based approach was used, no formal sample size calculation was performed. Although participation was voluntary, the high response rate minimises the likelihood of non‐response bias.

The study received approval from the Ondokuz Mayis University Clinical Research Ethics Committee (Approval No: 2022/384). All participants were informed about the study's nature and purpose and received standardised instructions before completing the questionnaire under supervision. Responses were collected immediately upon completion.

### 2.2 Questionnaire Design

The questionnaire was developed based on previously published studies addressing periodontal knowledge and oral health awareness, supplemented with original items relevant to the study objectives. The content was reviewed by faculty members with expertise in periodontology and biostatistics to ensure content clarity and relevance. The questionnaire was designed to assess baseline awareness, conceptual understanding and general educational outcomes related to periodontal health, rather than advanced clinical or specialist‐level knowledge.

The demographic information of participants, including gender, ethnicity, age and academic year, was collected at the beginning of the survey. The questionnaire consisted of 32 questions and was designed to assess medical students' knowledge, behaviours and perceptions related to periodontal disease. It was divided into four sections for focused analysis.

The first part was designed to evaluate oral hygiene practices, including 9 questions. Participants were asked about their oral hygiene behaviours: (1) which oral hygiene methods they use in their daily routine; (2) how often they brush their teeth; (3) how long they brush their teeth each time; (4) how often they use interdental cleaning tools; (5) whether they have ever visited a dentist; (6) the primary reasons for seeking dental care; (7) how they evaluate their overall gum health; (8) whether they have ever been diagnosed with periodontal disease by a dentist; and (9) when they last underwent professional dental scaling.

The second part focused on the inclusion of periodontal topics in the medical curriculum, assessed through five questions: (1) when students first received education on oral and dental health; (2) their primary sources of information about periodontal disease and its treatment; (3) whether they received any education on periodontal disease during medical school; (4) in which year this education took place; and (5) in which medical block it was included.

The third part assessed the knowledge of periodontal disease and compared the responses between first‐ and final‐year students. It included 5 questions: (1) what microbial dental plaque is; (2) what microbial dental plaque causes; (3) which statements best describe periodontal disease; (4) which clinical findings indicate periodontal disease; and (5) which systemic diseases or conditions are associated with periodontal diseases.

The final part of the questionnaire assessed students' overall attitudes and awareness regarding periodontal health, using 13 statements rated on a five‐point Likert scale (from ‘strongly agree’ to ‘strongly disagree’).

### 2.3 Statistical Analysis

Data were analysed using SPSS 21.0. Descriptive statistics were presented as mean ± standard deviation or percentages. The Mann–Whitney *U* test was used for continuous variables, while Pearson's Chi‐square test or Fisher's Exact test was applied for categorical variables. A *p*‐value less than 0.05 was considered to be statistically significant.

## 3 Results

A total of 599 questionnaires were distributed, and 596 completed responses were collected. The demographic characteristics of the subjects are presented in Table 1.

**TABLE 1:** Demographic characteristics of subjects participated in the survey (*n*).

Grade	Female	Male	Medical students
First‐year	145	136	281
Final‐year	173	142	315
Total			596

Gender, age and ethnicity were matched across groups with no statistically significant differences (data not shown).

### 3.1 Oral Hygiene Behaviour

As shown in Table 2, oral hygiene behaviours such as the use of additional oral hygiene aids and the frequency of daily tooth brushing were similar between first‐ and final‐year medical students. Likewise, the proportion of students who rated their gingival health as ‘good’ or better did not significantly differ between the groups. On the other hand, longer brushing durations were more commonly reported by first‐year students, whereas daily use of interdental cleaning tools and having undergone dental scaling within the past 6 months were more frequently observed among final‐year students, with all differences being statistically significant.

**TABLE 2:** Oral hygiene practices and periodontal care habits of medical students (%)^a^.

Question	First‐year	Final‐year	*p*
Oral hygiene aids with toothbrush	74.3	79.0	0.180
Frequency of daily tooth brushing (≥ twice)	76.5	79.7	0.372
Duration of tooth brushing (≥ 2 min)	61.9	52.7	**0.005**
Daily interdental cleaning	14.2	22.2	**0.022**
Self‐perceived gingival health (good or better)	66.5	65.9	0.657
Last dental scaling (< 6 months)	14.6	26.0	**< 0.001**

^a^ Pearson's Chi‐square test, bold numbers meant *p* < 0.05.

Regarding dental visits, 93.6% of first‐year and 96.2% of final‐year students reported having visited a dentist at least once, with no statistically significant difference between the groups (*p* = 0.148). Additionally, 11.7% of first‐year and 15.9% of final‐year students reported having been previously diagnosed with periodontal disease by a dentist, which was also not statistically significant (*p* = 0.146). The primary reasons for dental visits are presented in Figure 1, with dental caries being the most commonly reported concern among both first‐year and final‐year students.

**FIGURE 1:**
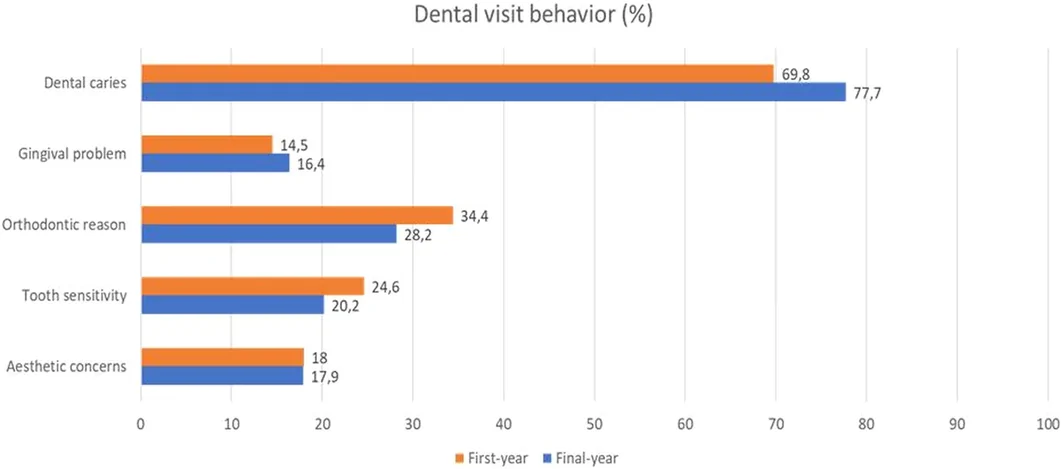
Primary reasons for dental visits among first‐ and final‐year medical students. Dental caries was the most commonly reported reason in both groups. Gingival complaints were the least frequently cited reason for seeking dental care.

### 3.2 Periodontal Education in the Medical Curriculum

When asked about the stage at which they first received education on oral and dental health, no statistically significant difference was found between first‐ and final‐year students (*p* = 0.088). The proportion of students who reported first receiving such education during undergraduate (medical school) years was 19.9% among first‐year students and 14.3% among final‐year students.

As illustrated in Figure 2, the most common sources of knowledge on periodontal disease were medical education and knowledge exchange with dentists. While final‐year students most frequently cited dentists and formal education as their primary sources, first‐year students relied more heavily on media platforms such as television, the internet and newspapers.

**FIGURE 2:**
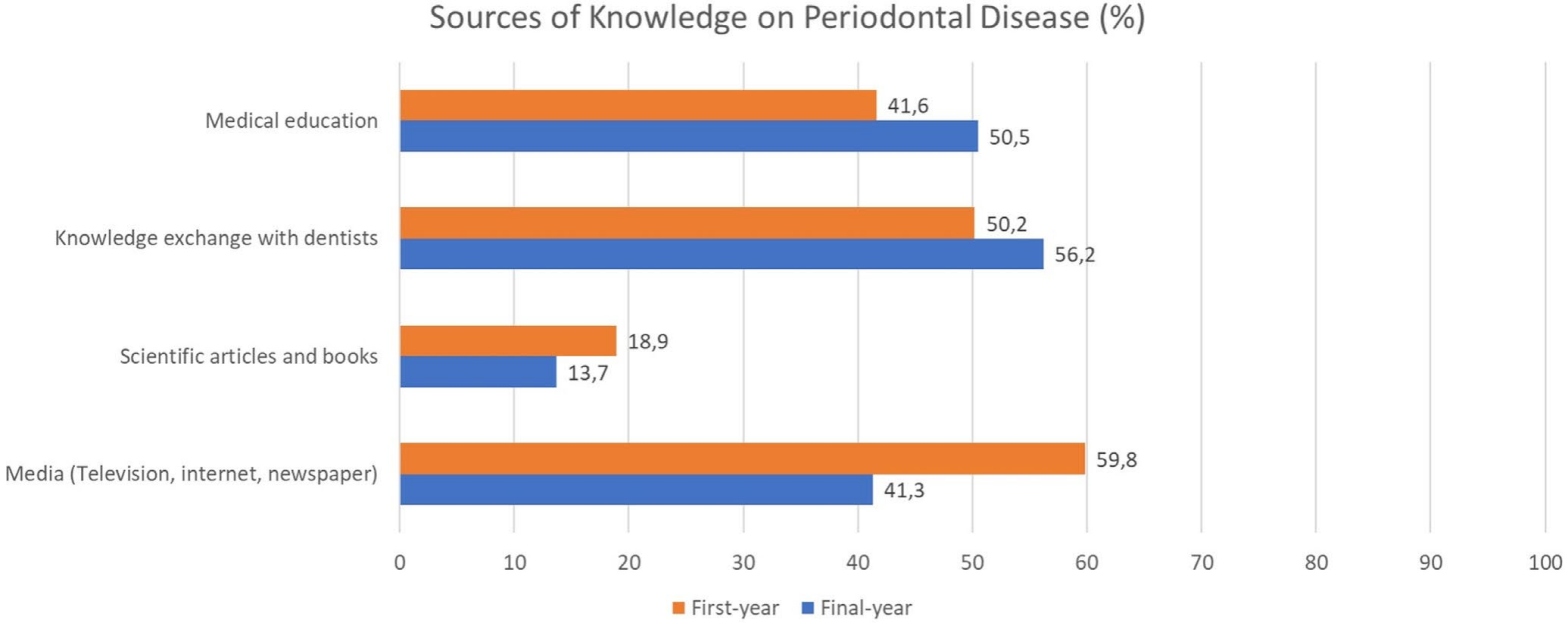
Sources of information on periodontal disease among medical students. Final‐year students most frequently reported obtaining information through knowledge exchange with dentists, whereas first‐year students most commonly accessed information via mass media sources such as television, internet and newspapers.

22.4% of first‐year and 41.3% of final‐year students reported receiving education on periodontal disease during medical school, with the majority of final‐year students (43.9%) indicating that this education was provided in the first year.

The distribution of educational blocks in which students reported receiving periodontal education is shown in Table 3. First‐year students most frequently cited the Multidisciplinary Practice block (90.0%) as the source of this education. Although not listed among the original response options, this block was often written in the free‐text ‘Other’ field and therefore coded separately. Among final‐year students, responses were more varied, with Otolaryngology (40.9%), Paediatrics (25.4%) and Internal Medicine (18.2%) most commonly selected. Notably, 22.7% of final‐year students also mentioned the Multidisciplinary Practice block.

**TABLE 3:** Periodontal diseases in the medical curriculum: Student perspectives (%).

Educational block	First‐year	Final‐year
Multidisciplinary Practice^a^	90.0	22.7
Other	10.0	9.1
Internal Medicine	—	18.2
Otolaryngology	—	40.9
Paediatrics	—	25.4

^a^ Multidisciplinary Practice refers to a first‐year educational block in the medical curriculum in which periodontal topics are introduced through applied teaching delivered by periodontists.

### 3.3 Knowledge of Periodontal Disease

In Table 4, five knowledge‐based questions were used to assess students' understanding of periodontal disease. The first two questions required students to select a single correct answer, while the remaining three allowed for multiple correct responses.

**TABLE 4:** Comparison of knowledge levels regarding periodontal disease between first‐year and final‐year medical students (%).

Question	First‐year	Final‐year	*p*
What is microbial dental plaque?			
I don't know	57.2	36.2	
Tooth discoloration	1.1	1.6	
Hard deposit on tooth surface	13.5	23.2	
Soft deposit on tooth surface	32.7	39.0	0.109
What does microbial dental plaque cause?			
I don't know	54.8	35.2	
Gingival disease	22.1	44.1	**< 0.001**
Tooth discoloration	20.3	16.2	
Tooth deformity	2.8	4.4	
Which of the following best describes periodontal diseases? (multiple choice)			
I—It is a disease characterised by inflammation and caused by multiple microorganisms	87.9	92.7	
II—It is an autoimmune disease	13.9	10.5	
III—It is an infection caused by a single type of microorganism	11.0	10.5	
IV—It is always accompanied by a tumoral process	3.9	2.2	
The correct answer: only I	74.7	80.3	0.102
Which of the following clinical findings suggest the presence of periodontal disease? (multiple choice)			
I—Gingival bleeding	73.0	86.7	
II—Tooth mobility	40.9	60.3	
III—Alveolar bone loss	43.8	48.9	
IV—Tooth loss	52.0	55.6	
V—Dental caries	49.5	41.6	
The correct answer: I, II, III and IV	4.6	15.9	**< 0.001**
Which of the following systemic diseases and conditions are associated with periodontal disease? (multiple choice)			
I—Diabetes	74.7	87.0	
II—Cardiovascular diseases	49.1	64.4	
III—Preterm birth	24.9	36.8	
IV—Low birth weight	22.4	38.4	
The correct answer: I, II, III and IV	11.4	27.6	**< 0.001**

*p* values were calculated using Pearson’s Chi‐square test.

When asked about the definition of microbial dental plaque, a considerable proportion of both groups selected the ‘I don't know’ option, with first‐year students choosing it more frequently. Although the overall distribution of responses differed significantly between the groups, the proportion of correct answers—defined as ‘soft deposit on the tooth surface’—was not statistically different (*p* = 0.109).

In contrast, final‐year students were significantly more likely to correctly identify the consequence of microbial plaque as gum disease. First‐year students more often reported uncertainty. Both the overall response distribution and the accuracy of the answers showed statistically significant differences in favour of final‐year students (*p* < 0.001).

For the third knowledge question, which asked students to identify the correct definition of periodontal disease, the accurate response was selecting only ‘a disease characterized by inflammation and influenced by multiple microorganisms.’ The proportion of correct answers did not differ significantly between the two groups (*p* = 0.102), with 74.7% of first‐year and 80.3% of final‐year students answering correctly.

For the fourth knowledge question, students were asked to identify which clinical findings suggest the presence of periodontal disease. The correct answer included gingival bleeding, tooth mobility, alveolar bone loss and tooth loss. While most participants selected at least one correct option, only a small proportion of students chose all four correct answers. Notably, final‐year students (15.9%) were significantly more likely than first‐year students (4.6%) to provide the fully correct response (*p* < 0.001), indicating a statistically significant difference in diagnostic knowledge.

Final‐year students demonstrated significantly better awareness regarding the systemic associations of periodontal disease. The proportion of students who correctly identified all relevant conditions—diabetes, cardiovascular diseases, preterm birth and low birth weight—was notably higher among final‐year students compared to first‐year students (*p* < 0.001).

### 3.4 Attitudes and Awareness Toward Periodontal Health

Students' attitudes and awareness regarding periodontal health were assessed through 13 Likert‐scale statements, and the response distributions are presented in Table 5.

**TABLE 5:** Attitudes and awareness toward periodontal health among medical students (%)^a^.

Question	First‐year	Final‐year	*p*
Periodontal health is important for general health.	88.3	89.8	0.164
There is a strong relationship between periodontal disease and systemic health.	81.1	86.0	0.064
Periodontal disease is an important condition that needs treatment.	89.3	89.9	0.232
Plaque is a significant factor in gingivitis.	49.5	62.9	**< 0.001**
Daily brushing is essential to maintain periodontal health.	85.0	90.1	0.075
There is an evidence‐based strong link between periodontal disease and diabetes.	47.3	72.1	**< 0.001**
I am informed about current studies on the relationship between periodontal disease and diabetes.	23.1	35.2	**0.008**
Diabetic patients are at high risk for severe periodontal disease.	47.7	76.5	**< 0.001**
Healthcare professionals should be informed about periodontal disease.	85.4	83.2	0.965
Dentists and clinicians should cooperate to reduce the risk of periodontal disease.	86.5	88.9	0.449
I believe I can perform oral health examinations for my patients.	47.7	45.4	0.161
I want to improve my knowledge in oral health education and examination.	77.9	75.9	0.181
I need additional information about the impact of periodontal disease on systemic diseases.	83.3	79.7	0.316

*Note:* Values indicate the combined percentage of participants who responded ‘Agree’ and ‘Strongly Agree’ to each statement.

^a^ Pearson's Chi‐square test, bold numbers meant *p* < 0.05.

Overall, both first‐year and final‐year medical students demonstrated high awareness regarding the importance of periodontal health and its relevance to general health. However, significant differences were observed between the groups in specific areas, particularly in the understanding of the relationship between periodontal disease and diabetes, and the role of plaque in gingivitis (*p* < 0.001).

## 4 Discussion

Periodontal diseases are prevalent chronic inflammatory conditions that not only affect oral health but also have well‐established associations with systemic health, underscoring the need for collaboration between dentists and physicians as part of an integrated approach to public health [13, 16]. Hassona et al. compared final‐year medical and dental students in terms of their ability to recognise oral manifestations of systemic diseases, reporting that medical students lacked adequate knowledge, diagnostic ability and confidence [17]. Similarly, Yao et al. assessed the oral health knowledge and behaviours of first‐ and third‐year medical and dental students in China, and emphasised the need for improved cooperation between medical and dental education, along with curriculum reform [18]. Based on these findings, this study assessed the knowledge, attitudes and behaviours related to periodontal disease among first‐ and final‐year medical students at a public medical school. Our results showed that final‐year students had significantly better knowledge compared to first‐year students, indicating a positive impact of medical education. However, the overall performance on knowledge‐based questions was poor across both groups, revealing critical deficiencies in understanding periodontal disease.

Analysis of the knowledge‐based questions indicates that students' understanding of periodontal–systemic relationships varies depending on the specific condition assessed. The association between periodontal disease and diabetes was relatively well recognised, with 74.7% of first‐year students and 87.0% of final‐year students correctly identifying this link. These findings show notable parallels with those reported by Owens et al., who examined physicians' understanding of the periodontal–diabetes relationship and found that approximately 78% recognised the increased risk of periodontitis in diabetic patients [15]. However, despite this general awareness, knowledge of the scientific basis underlying this association remained limited. In Owens et al.'s study, 40% of physicians were unfamiliar with the supporting literature, while in the present study only 35.2% of final‐year medical students reported awareness of current research on the topic. In contrast, awareness of other systemic consequences of periodontal disease was markedly lower. Less than half of the students identified associations with adverse pregnancy outcomes, including preterm birth and low birth weight, a pattern consistent with previous findings reported by Parveen et al. [19]. Together, these results suggest that medical education may emphasise selected periodontal–systemic links, such as diabetes, while underrepresenting others, leading to uneven knowledge distribution even at the point of graduation. This educational emphasis is consistent with previous studies showing that periodontal–systemic relationships such as diabetes, cardiovascular diseases and adverse pregnancy outcomes are more frequently highlighted in medical and interprofessional education contexts, whereas broader systemic associations receive less attention [15, 18].

Our findings revealed two critical issues in students' understanding of periodontal disease. First, a significant proportion of students confused dental caries with periodontal clinical signs. Specifically, 49.5% of first‐year and 41.6% of final‐year students in our sample incorrectly identified dental caries as a symptom of periodontal disease, suggesting a fundamental misunderstanding of the basic differences between the two conditions. In contrast, only 9.5% of participants in the study by Cohen et al., which included practicing gynaecologists, made this error [20]. Although these physicians were not dental professionals, they demonstrated a clearer conceptual distinction between caries and periodontal disease compared to our medical student sample. The persistence of this misconception may be partly explained by limited exposure to structured oral pathology education within the medical curriculum and the predominance of caries‐focused public health messaging, which may lead students to associate most oral disease symptoms with tooth decay rather than periodontal pathology. In addition, insufficient emphasis on the etiopathogenesis of periodontal disease may hinder students' ability to differentiate between diseases with distinct biological mechanisms. Second, although gingival bleeding was widely recognised as a clinical indicator (86.7% among our final‐year students), recognition of other key signs of disease progression showed a noticeable decline. A similar trend was observed in Cohen's study, where gingival bleeding was the most commonly identified symptom (87.4%), while awareness of later‐stage findings such as bone loss and tooth loss was considerably lower. These patterns suggest that medical education may focus predominantly on early or visible manifestations of oral disease, while providing limited instruction on disease progression and long‐term clinical consequences. Together, these findings highlight the need for more conceptually structured and progression‐oriented periodontal education within medical training.

Interestingly, while only 47.7% of first‐year and 45.4% of final‐year students felt confident in their ability to perform oral health examinations, a markedly higher proportion expressed a desire to improve their knowledge in oral health education and examination (77.9% and 75.9%). This contrast highlights a significant gap between students' motivation and their perceived clinical competence. This gap between motivation and self‐confidence is especially concerning given the well‐documented links between oral health and systemic conditions. For example, Summers et al. emphasised that proper oral examination in patients with renal disease plays a vital role in preventing complications related to both the underlying condition and the therapies used in its management, illustrating the broader medical relevance of oral health competencies [21]. As Chan et al. argue, the historical separation of medicine and dentistry has led to a lack of collaboration between the two fields; however, the widespread burden of chronic diseases with both medical and dental implications necessitates a more integrated and interdisciplinary approach to healthcare [22].

A recent pilot study by Morel et al. in the United States demonstrated that a structured periodontal training program, integrating both theoretical instruction and clinical practice, significantly enhanced third‐year medical students' knowledge and confidence in oral examination and patient counselling [23]. In contrast, at the participating institution in the present study, periodontal topics are primarily introduced during a first‐year multidisciplinary practice block taught by periodontologists. Although this early exposure may support basic awareness, the persistent knowledge gaps observed among final‐year students suggest that such isolated curricular components do not provide sufficient reinforcement over time. Taken together with the observed differences between first‐year and final‐year students, these findings indicate that while medical education contributes to incremental improvements in periodontal knowledge, the level attained by graduation remains inadequate. This mismatch between curricular exposure and achieved competency points to a fragmented educational structure that lacks longitudinal integration. From a practice domain perspective, limited and non‐recurrent clinical exposure to periodontal assessment during medical training may restrict students' ability to translate theoretical knowledge into routine clinical decision‐making, underscoring the need for more integrated and practice‐oriented periodontal education within medical curricula.

This study has some limitations that should be acknowledged. Conducted at a single medical school, the findings may not be fully generalizable to other institutions or educational systems, although the curriculum aligns closely with national standards. The cross‐sectional design limits insights into longitudinal knowledge development and self‐reported data may be influenced by social desirability bias, potentially leading to an overestimation of knowledge or attitudes. Although the questionnaire was informed by existing literature and reviewed by experts, it was not psychometrically validated, which may impact the reliability of the results. In addition, academic performance indicators such as grade point average (GPA) were not assessed and may have influenced knowledge levels. Furthermore, potential confounding factors such as socioeconomic background, prior exposure to dental education or individual interest in oral health were not fully accounted for, and future studies using validated instruments and multivariate approaches are recommended to address these issues.

## 5 Conclusion

In this study comparing first‐year and final‐year medical students, final‐year participants demonstrated higher levels of knowledge regarding periodontal disease. The findings suggest that progression through medical education contributes to improved awareness of periodontal health and its systemic associations. However, the overall knowledge levels in both groups were found to be insufficient. Although many students felt inadequately informed, a high level of willingness to improve their knowledge was observed. These results highlight the importance of integrating structured periodontal education into undergraduate medical curriculum to support early diagnosis, appropriate referrals and prevention of periodontal‐systemic complications. Accordingly, enhancing periodontal teaching within medical school curricula through structured, interdisciplinary and clinically relevant educational approaches may help bridge existing knowledge gaps and strengthen oral–systemic health competence among future physicians.

## Funding

The authors have nothing to report.

## Conflicts of Interest

The authors declare no conflicts of interest.

## Data Availability

The data that support the findings of this study are available from the corresponding author upon reasonable request.

## References

[1] D. F. Kinane , “Periodontal Diagnostics,” Annals of the Royal Australasian College of Dental Surgeons 15 (2000): 34–41. 11709970

[2] J. Kim and S. Amar , “Periodontal Disease and Systemic Conditions: A Bidirectional Relationship,” Odontology 94, no. 1 (2006): 10–21, 10.1007/s10266-006-0060-6. PMC2443711 16998613

[3] P. Chen , F. Hong , and X. Yu , “Prevalence of Periodontal Disease in Pregnancy: A Systematic Review and Meta‐Analysis,” Journal of Dentistry 125 (2022): 104253, 10.1016/j.jdent.2022.104253. 35998741

[4] L. Nibali , N. Gkranias , G. Mainas , and A. Di Pino , “Periodontitis and Implant Complications in Diabetes,” Periodontol 2000 90, no. 1 (2022): 88–105, 10.1111/prd.12451. PMC9805043 35913467

[5] C. Z. Wu , Y. H. Yuan , H. H. Liu , et al., “Epidemiologic Relationship Between Periodontitis and Type 2 Diabetes Mellitus,” BMC Oral Health 20, no. 1 (2020): 204, 10.1186/s12903-020-01180-w. PMC7353775 32652980

[6] M. Sanz , A. Marco Del Castillo , S. Jepsen , et al., “Periodontitis and Cardiovascular Diseases: Consensus Report,” Journal of Clinical Periodontology 47, no. 3 (2020): 268–288, 10.1111/jcpe.13189. PMC7027895 32011025

[7] M. A. Curtis , P. I. Diaz , and T. E. Van Dyke , “The Role of the Microbiota in Periodontal Disease,” Periodontol 2000 83, no. 1 (2020): 14–25, 10.1111/prd.12296. 32385883

[8] J. Jin , X. Zhang , Z. Lu , et al., “Simvastatin Inhibits Lipopolysaccharide‐Induced Osteoclastogenesis and Reduces Alveolar Bone Loss in Experimental Periodontal Disease,” Journal of Periodontal Research 49 (2014): 518–526, 10.1111/jre.12132. PMC3979522 24117880

[9] N. Delaleu and M. Bickel , “Interleukin‐1β and Interleukin‐18: Regulation and Activity in Local Inflammation,” Periodontology 2000 35, no. 1 (2004): 42–52, 10.1111/j.0906-6713.2004.003569.x. 15107057

[10] S. A. Hienz , S. Paliwal , and S. Ivanovski , “Mechanisms of Bone Resorption in Periodontitis,” Journal of Immunology Research 2015 (2015): 615486, 10.1155/2015/615486. PMC4433701 26065002

[11] H. Zhang , L. Liu , C. Jiang , K. Pan , J. Deng , and C. Wan , “MMP9 Protects Against LPS‐Induced Inflammation in Osteoblasts,” Innate Immunity 26, no. 4 (2020): 259–269, 10.1177/1753425919887236. PMC7251795 31726909

[12] J. L. Ebersole , J. Stevens , M. J. Steffen , et al., “Systemic Endotoxin Levels in Chronic Indolent Periodontal Infections,” Journal of Periodontal Research 45, no. 1 (2010): 1–7, 10.1111/j.1600-0765.2008.01169.x. PMC4102883 20465752

[13] M. A. Baseer , M. S. Alenazy , M. Alasqah , et al., “Oral Health Knowledge, Attitude and Practices Among Health Professionals in King Fahad Medical City, Riyadh,” Dental Research Journal (Isfahan) 9, no. 4 (2012): 386–392. PMC3491323 23162577

[14] W. E. Mouradian , A. Reeves , S. Kim , et al., “An Oral Health Curriculum for Medical Students at the University of Washington,” Academic Medicine 80, no. 5 (2005): 434–442, 10.1097/00001888-200505000-00004. 15851452

[15] J. B. Owens , R. S. Wilder , J. H. Southerland , J. B. Buse , and R. M. Malone , “North Carolina Internists' and Endocrinologists' Knowledge, Opinions, and Behaviors Regarding Periodontal Disease and Diabetes: Need and Opportunity for Interprofessional Education,” Journal of Dental Education 75, no. 3 (2011): 329–338. 21368257

[16] S. Rabiei , S. Z. Mohebbi , K. Patja , and J. I. Virtanen , “Physicians' Knowledge of and Adherence to Improving Oral Health,” BMC Public Health 12 (2012): 855, 10.1186/1471-2458-12-855. PMC3503732 23046660

[17] Y. Hassona , N. A. Salim , N. Tarboush , et al., “Knowledge About Oral Manifestations of Systemic Diseases Among Medical and Dental Students From Jordan: An Interdisciplinary Educational Gap,” Special Care in Dentistry 42, no. 4 (2022): 383–389, 10.1111/scd.12692. 34984709

[18] K. Yao , Y. Yao , X. Shen , C. Lu , and Q. Guo , “Assessment of the Oral Health Behavior, Knowledge and Status Among Dental and Medical Undergraduate Students: A Cross‐Sectional Study,” BMC Oral Health 19, no. 1 (2019): 26, 10.1186/s12903-019-0716-6. PMC6352450 30696440

[19] S. Parveen , A. S. Al Qahtani , E. Halboub , et al., “Periodontal‐Systemic Disease: A Study on Medical Practitioners' Knowledge and Practice,” International Dental Journal 73, no. 6 (2023): 854–861, 10.1016/j.identj.2023.05.003. PMC10658429 37330312

[20] L. Cohen , M. Schaeffer , J. L. Davideau , H. Tenenbaum , and O. Huck , “Obstetric Knowledge, Attitude, and Behavior Concerning Periodontal Diseases and Treatment Needs in Pregnancy: Influencing Factors in France,” Journal of Periodontology 86, no. 3 (2015): 398–405, 10.1902/jop.2014.140371. 25427617

[21] S. A. Summers , W. M. Tilakaratne , F. Fortune , and N. Ashman , “Renal Disease and the Mouth,” American Journal of Medicine 120, no. 7 (2007): 568–573, 10.1016/j.amjmed.2006.12.007. 17602925

[22] S. Chan , G. M. Pasternak , and M. J. West , “The Place of Periodontal Examination and Referral in General Medicine,” Periodontol 2000 74, no. 1 (2017): 194–199, 10.1111/prd.12199. 28429485

[23] M. M. Morel , E. Chuang , and N. Laniado , “Bridging Gaps in Oral Health Education in a Medical School in the United States: A Pilot Study,” BMC Medical Education 22, no. 1 (2022): 578, 10.1186/s12909-022-03648-5. PMC9330983 35902912

